# TRIM25 inhibits influenza A virus infection, destabilizes viral mRNA, but is redundant for activating the RIG-I pathway

**DOI:** 10.1093/nar/gkac512

**Published:** 2022-06-23

**Authors:** Nila Roy Choudhury, Ivan Trus, Gregory Heikel, Magdalena Wolczyk, Jacek Szymanski, Agnieszka Bolembach, Rute Maria Dos Santos Pinto, Nikki Smith, Maryia Trubitsyna, Eleanor Gaunt, Paul Digard, Gracjan Michlewski

**Affiliations:** Infection Medicine, University of Edinburgh, The Chancellor's Building, Edinburgh, UK; Dioscuri Centre for RNA-Protein Interactions in Human Health and Disease, International Institute of Molecular and Cell Biology in Warsaw, Warsaw, Poland; Infection Medicine, University of Edinburgh, The Chancellor's Building, Edinburgh, UK; The Wellcome Centre for Cell Biology, University of Edinburgh, Edinburgh, UK; Dioscuri Centre for RNA-Protein Interactions in Human Health and Disease, International Institute of Molecular and Cell Biology in Warsaw, Warsaw, Poland; Dioscuri Centre for RNA-Protein Interactions in Human Health and Disease, International Institute of Molecular and Cell Biology in Warsaw, Warsaw, Poland; Dioscuri Centre for RNA-Protein Interactions in Human Health and Disease, International Institute of Molecular and Cell Biology in Warsaw, Warsaw, Poland; The Roslin Institute, Easter Bush, University of Edinburgh, Edinburgh, UK; The Roslin Institute, Easter Bush, University of Edinburgh, Edinburgh, UK; Institute of Quantitative Biology, Biochemistry and Biotechnology, University of Edinburgh, Roger Land Building, Edinburgh, UK; The Roslin Institute, Easter Bush, University of Edinburgh, Edinburgh, UK; The Roslin Institute, Easter Bush, University of Edinburgh, Edinburgh, UK; Dioscuri Centre for RNA-Protein Interactions in Human Health and Disease, International Institute of Molecular and Cell Biology in Warsaw, Warsaw, Poland; Infection Medicine, University of Edinburgh, The Chancellor's Building, Edinburgh, UK

## Abstract

The E3 ubiquitin ligase TRIM25 is a key factor in the innate immune response to RNA viruses. TRIM25 has been shown to play a role in the retinoic-acid-inducible gene-1 (RIG-I) pathway, which triggers expression of type 1 interferons upon viral infection. We and others have shown that TRIM25 is an RNA-binding protein; however, the role of TRIM25 RNA-binding in the innate immune response to RNA viruses is unclear. Here, we demonstrate that influenza A virus (IAV A/PR/8/34_NS1(R38A/K41A)) infection is inhibited by TRIM25. Surprisingly, previously identified RNA-binding deficient mutant TRIM25ΔRBD and E3 ubiquitin ligase mutant TRIM25ΔRING, which lack E3 ubiquitin ligase activity, still inhibited IAV replication. Furthermore, we show that in human-derived cultured cells, activation of the RIG-I/interferon type 1 pathway mediated by either an IAV-derived 5′-triphosphate RNA or by IAV itself does not require TRIM25 activity. Additionally, we present new evidence that instead of TRIM25 directly inhibiting IAV transcription it binds and destabilizes IAV mRNAs. Finally, we show that direct tethering of TRIM25 to RNA is sufficient to downregulate the targeted RNA. In summary, our results uncover a potential mechanism that TRIM25 uses to inhibit IAV infection and regulate RNA metabolism.

## INTRODUCTION

Influenza A virus (IAV) is a ubiquitous human pathogen that causes seasonal epidemics and sporadic pandemics, the most recent of which was in 2009. Seasonal IAV kills up to 500 000 people annually, thereby causing a significant global socioeconomic burden (1). Vaccines are updated yearly to target contemporary strains, but this relies on making predictions about an unpredictable virus. Alternative interventions targeting host cell factors are attracting increasing interest to overcome the issues forthcoming due to high viral diversity. To identify such targets, it is essential to understand how viruses interact with host cell factors.

The innate immune system is the body's first line of defence against viral infection. Viruses may be detected by the host cell, triggering signalling cascades that result in the expression of interferon proteins and antiviral interferon-stimulated genes (2,3). E3 ubiquitin ligase TRIM25 (a member of the tripartite motif (TRIM) family of proteins) has emerged as a key factor in triggering the innate immune response to RNA viruses (4,5). All TRIMs have in common their amino-terminal tripartite domain arrangement—RING–Bbox1/2–coiled coil (CC)—yet differ in their C-terminal domains, informing their characterization into several subtypes. TRIM25 has been shown to play a role in the retinoic-acid-inducible gene-1 (RIG-I) pathway, which triggers expression of type I interferons upon viral infection (6). RIG-I binds to RNA molecules with a 5′-triphosphate (5′ppp) moiety that are produced during viral replication (7–9). This provokes a RIG-I conformational change allowing TRIM25 multimers to bind to and ubiquitinate the RIG-I tandem caspase recruitment domains (2CARD), thus triggering a series of events that culminate in the phosphorylation of IRF-3, IRF-7 and NF-κB. These transcription factors then translocate to the nucleus and induce type I interferon expression, leading to an innate immune response (10–12). There is also evidence that RIG-I signalling can be activated by unanchored K63-linked polyubiquitin chains that are generated by TRIM25 (13). Recent data however suggests that TRIM25 may not play a key role in RIG-I activation as previously proposed (14,15). These studies revealed that another E3 ubiquitin ligase RIPLET (RNF135), not TRIM25, is sufficient to ubiquitinate and activate the RIG-I. This discrepancy could have arisen from studying TRIM25′s role in the context of the isolated RIG-I 2CARD domain, which is readily ubiquitinated by TRIM25 (6).

We and others have revealed that TRIM25 is an RNA-binding protein (RBP) (16–21). TRIM25 binds RNAs (either single- or double-stranded) through an RNA-binding domain (RBD) residing in its C-terminal PRY/SPRY region (16). RNA-binding appears to be crucial for its E3 ubiquitin ligase activity as deletion of the RNA-binding domain reduces TRIM25 ubiquitination *in vitro* (16). Additional TRIM25′s RNA-binding domains have been found, highlighting biochemical complexity of this newly identified RBP (20,22). However, specific sequences or conformational RNA motifs which attract TRIM25 binding have not been identified.

Consistent with a role in antiviral sensing, TRIM25 is known to be inhibited by several RNA viruses, resulting in dampening of the innate immune response (19,23–25). For IAV, this is via the non-structural protein 1 (NS1). NS1 directly binds to the coiled-coil domain of TRIM25, disrupting its dimerization and consequently, its ubiquitin ligase activity (23,26). However, although NS1 proteins from several IAV strains bind to TRIM25, not all of these are capable of inhibiting phosphorylation of IRF-3 (presumably following RIG-I activation) upon virus infection (27–29). This indicates that NS1, among its many other roles, may be targeting an alternative function of TRIM25. Additionally, NS1 can also bind to and antagonise TRIM25 function in chicken cells, where no RIG-I orthologue has been identified, hinting at a RIG-I-independent antiviral function for TRIM25 (27). Indeed, recent work has suggested that TRIM25 can restrict IAV by binding to viral ribonucleoproteins (RNPs) and inhibiting viral mRNA chain elongation (30). This interaction is RNA-dependent, involving direct binding of TRIM25 to the IAV genomic vRNA, though a detailed map of where is lacing. Nevertheless, an IAV A/PR/8/34 (PR8) strain with R38A/K41A (R38K41A) mutations in NS1 is sensitive to TRIM25, providing a good platform to study TRIM25′s functions (23,31–34).

Here, we confirm that in human-derived HEK293 cells, IAV replication is sensitive to TRIM25 using a virus with NS1 protein mutant R38K41A. However, inhibition of viral replication by TRIM25 did not require TRIM25 E3 ubiquitin ligase activity or the PRY/SPRY RNA binding domain, nor did it correlate with the ability of virally derived 5′-pppRNA or PR8 R38K41A IAV to stimulate the RIG-I/IRF-3 pathway. Furthermore, the inhibitory function of TRIM25 did not appear to work by direct inhibition of viral transcription. Using RNA-seq and CLIP-seq analyses we found that TRIM25 binds promiscuously to positive strand IAV RNAs, whereas the binding to negative strand RNAs was much weaker and confined to segment ends. Our results confirm that TRIM25 is not necessary for RIG-I activation in human cultured cells and uses alternative, RIG-I-independent mechanisms in restricting IAV infection. Indeed, we identify a novel function of TRIM25 which triggers destabilization of IAV mRNAs. Crucially, direct tethering of TRIM25-MS2 fusion protein to MS2 stem loops integrated into the 3′UTR of the luciferase mRNA significantly reduced luciferase mRNA levels. These results shed light on TRIM25 in the innate immune response to RNA viruses and present evidence for TRIM25′s role in regulation of viral mRNA metabolism.

## MATERIALS AND METHODS

### Virus infections

Influenza virus strain A/Puerto Rico/8/1934 H1N1 and its NS1 mutant (R38K41A) were used for infections and were generated by reverse genetics using plasmids encoding all 8 segments of the IAV genome (31,35).

To evaluate viral replication kinetics, HEK293 cells were inoculated in suspension at an MOI of 0.0001 in 300 μl of DMEM supplemented with 0.14% BSA. Cells were then seeded in 12-well plates to obtain a resulting concentration of 2 × 10^6^ cells per well. Mock-infected cells were included as controls. Cells were incubated with virus (5% CO_2_, 37°C) for 3 h before the addition of 2 ml of virus growth medium (DMEM, 0.14% BSA, 0.5 μg/ml TPCK-treated trypsin). Supernatants were collected with three technical replicates every 24 h and frozen (−80°C) until subsequent infectious virus quantification with the endpoint dilution assay (36). Fifty percent cell culture infective dose (CCID_50_) endpoint titers were calculated by the Spearman–Kärber formula and expressed as a decimal logarithm. Media from mock-inoculated cells were used as negative controls.

For Favipiravir treatments, HEK293 cells were seeded in six-well plates such that they reached a confluency of 70% before being infected with virus at an MOI of 0.01. Cells were incubated with virus diluted in DMEM for 0.5 h. Then inoculum was replaced with virus growth medium (DMEM, 0.14% BSA, 1 μg/ml TPCK-treated trypsin). After 1.5 h, medium was replaced with cell culture medium (DMEM, 10% FBS) supplemented with 500 μM of Favipiravir (Selleck Chemicals) in DMSO. Control cells were incubated in cell culture medium with 0.5% DMSO added. The cells were incubated for indicated times and then cells were re-suspended in TRI Reagent (Sigma-Aldrich).

### CLIP-seq

HEK293 TRIM25 KO cells with integrated T7-tagged TRIM25 WT or TRIM25ΔRBD were infected with the IAV PR8 R38K41A strain at an MOI of 5 for 6 h followed by UV crosslinking. CLIP-seq was then performed as described previously (16).

The part CLIP is a variation of our previously published CLIP-seq method where the RNA bound by the target protein is radiolabelled for visualization. Briefly, two P100 dishes per sample of HEK293 TRIM25 KO cells transfected with plasmids expressing T7-tagged TRIM25 WT, TRIM25ΔRBD or GFP over night were UV-crosslinked. Anti-T7 antibody (Merck Millpore, #69522) was coupled to 30 μl of Dynabeads, the T7-tagged protein-RNA complexes immunoprecipitated and the RNA partially digested using RNase A and radiolabelled using γ^32^P-ATP for 20 min at 37°C. The beads were boiled at 70°C in NuPAGE LDS sample buffer (Invitrogen) and the supernatant loaded on to a 4–12% Bis–Tris NuPAGE SDS-PAGE gel (Invitrogen) and protein-RNA complexes transferred onto a nitrocellulose membrane and exposed to film.

Raw sequence data was de-multiplexed (cutadapt v. 3.5), trimmed (Trimmomatic v. 0.39; parameters: ‘LEADING:3 TRAILING:3 SLIDINGWINDOW:4:15 MINLEN:16’), and checked with Kallisto v. 0.46.1 for strand direction. Kallisto was used to build an index of coding and non-coding RNAs from the host (Ensembl human gene annotation version GRCh38.105) and to quantify reads mapped to the host transcriptome. IAV reference genome for the PR8 R38K41A IAV was *de novo* assembled (bowtie2 v. 2.4.2 and UGENE v. 40.1) and used as a reference for mapping viral reads upon removal of duplicated reads (FASTX-Toolkit v. 0.0.13 and bowtie2). Coverage was assessed separately for positive- and negative-sense reads (samtools v. 1.15 and SeqKit v. 2.1.0). The accession number for CLIP-seq data is PRJNA810715 in NCBI BioProject.

### RNA-seq

HEK293 TRIM25 KO cells with integrated T7-tagged TRIM25-WT or TRIM25ΔRBD cells (*n* = 3) were infected with the PR8 R38K41A IAV for 6 h at an MOI of 5. Total RNA from cells was extracted with TRI Reagent. RNA samples were quantified using Qubit 2.0 Fluorometer (Invitrogen, Carlsbad, CA, USA) and RNA integrity was checked using Agilent Fragment Analyzer (Agilent Technologies, Palo Alto, CA, USA). After rRNA depletion (NEBNext rRNA Depletion Kit) and stranded RNA sequencing library preparation (NEBNext Ultra II Directional RNA Library Prep Kit). Briefly, enriched RNAs were fragmented for 15 min at 94°C. First strand and second strand cDNAs were subsequently synthesized. cDNA fragments were end repaired and adenylated at 3′ ends, and universal adapters were ligated to cDNA fragments, followed by index addition and library enrichment by limited-cycle PCR. The sequencing libraries were validated on the Agilent Fragment Analyzer (Agilent Technologies, Palo Alto, CA, USA), quantified by using Qubit 2.0 Fluorometer (Invitrogen, Carlsbad, CA, USA), and subjected to paired-end stranded sequencing with 150 nt reads (Illumina NovaSeq 6000). Raw sequence data was de-multiplexed (bcl2fastq v. 2.20), trimmed (Trimmomatic v. 0.39; parameters: ‘LEADING:3 TRAILING:3 SLIDINGWINDOW:4:15 MINLEN:16’), and checked with Kallisto for strand direction. Kallisto was used to build an index of coding and non-coding RNAs from the host (Ensembl human gene annotation version GRCh38.105) and to quantify reads mapped to the host transcriptome. IAV reference genome for the PR8 R38K41A IAV was *de novo* assembled (bowtie2 and UGENE) and used as a reference for mapping viral reads upon removal of duplicated reads (Nubeam-dedup and bowtie2). Coverage was assessed separately for positive- and negative-sense reads (samtools v. 1.15 and SeqKit v. 2.1.0). The accession number for RNA-seq data is PRJNA810715 in NCBI BioProject.

### 3p-hpRNA assays

Cell lines were transfected at 70% confluency with 100 ng/ml 3p-hpRNA (Invivogen) or dephosphorylated with alkaline phosphatase (Thermo Scientific, #EF0654) form of 3p-hpRNA using Lipofectamine 2000. For western blots, cells were incubated for 6–24 h before protein extraction. 80 μg protein lysate was loaded per well. Proteins were detected using the following antibodies and dilutions: phospho-IRF-3 (Rabbit mAb, Cell Signalling Technologies, 1:1000), IRF-3 (Rabbit mAb, Cell Signalling Technologies, 1:1000 or Rabbit pAb, Protein-Tech, 1:1000), RIG-I (Rabbit mAb, Cell Signalling Technologies, 1:1000), TRIM25 (Rabbit pAb, Abcam, 1:2000), DHX9 (Rabbit pAb, Protein-Tech, 1:1000) and alpha tubulin (Rabbit pAb, Protein-Tech, 1:5000). For HEK-Blue assays, supernatants were harvested 6 h post-transfection. 20 μl of the supernatant or an IFNα/β standard containing purified IFNα and IFNβ were added to 180 μl HEK-Blue cells (Invivogen) (50,000 cells) and incubated for 24 h. Five microliter of this supernatant was added to 180 μl QUANTI-Blue (Invivogen) and incubated for 1 h at 37°C before absorbance was read at 680 nm. For luciferase assays, cells were transfected with 100 ng/ml 3p-hpRNA along with a Firefly luciferase reporter under the IFNβ promoter and Renilla luciferase under a constitutive reporter. The cells were incubated for 24 h post-transfection before extraction with passive lysis buffer (Promega). Activity levels were measured for Firefly and Renilla luciferase using the Dual-Luciferase Reporter Assay System (Promega) according to the manufacturer's instructions.

### HEK293 cells CRISPR/Cas9 knockdown of RIG-I

HEK293 cells were co-transfected with 200 ng of GeneArt CRISPR Nuclease mRNA (Invitrogen, #A29378) in addition to sgRNA prepared by mixing Alt-R CRISPR-Cas9 tracrRNA (IDT, #1072532) with Alt-R CRISPR-Cas9 crRNA targeting sequence in intron 1 of DDX58 gene encoding RIG-I protein (IDT, #Hs.Cas9.DDX58.1.AA – GGAUUAUAUCCGGAAGACCC) at a 4 nM final concentration. After 24 h, cells were subcloned by the limiting dilution technique, and the cells were grown until single colonies were established. Next, cells were split into two 96-well plates, one of which was used for a dot blot analysis. For the dot blot analysis, cells were washed once in cold PBS prior to the addition of 20 μl of Roeder D Lysis Buffer per well and sonication for 10 min (30 s ON/30 s OFF). Six microliters of protein from each well were spotted directly onto a nitrocellulose membrane followed by western blotting. Selected clones were seeded from the second 96-well plate into six-well plates, grown, and RIG-I levels were validated by standard western blotting. Recombinant human IFNβ (R&D Systems, #8499-IF-010) treatment was used to induce RIG-I expression and to correctly select clones with the lowest RIG-I level. This strategy led to creation of several HEK293 cell clones with partial depletion of RIG-I expression (Supplementary Figure S5). For further analysis, we selected clone E7 (Supplementary Figure S5) with a partial RIG-I depletion and clone F5 (Supplementary Figure S5) with almost complete RIG-I knockdown.

### Calu-1 cell Crispr/Cas9 knockdown of TRIM25

Knock-out of TRIM25 in Calu-1 cells (a gift from Dr Christine Tait-Burkard at the University of Edinburgh, purchased from ECACC) was performed as described for RIG-I. A guide RNA (CAACGGGGCGTCGAGAGCAC) targeting the second exon in TRIM25 pre-designed and purchased from IDT (Alt-R CRISPR-Cas9 crRNA XT) was used. Genotyping was performed using a pair of primers amplifying the guide target area (forward: AATCCTCCCAATAGCCCTGT, reverse: ACAGCTGTCTGCATGCTTTG).

### IAV minireplicon assay

Cells were transfected with plasmids encoding segments 1, 2, 3 and 5 (PA, PB1, PB2, NP) of the IAV genome (31). For the luciferase reporter assay, a plasmid encoding Firefly luciferase that is transcribed as a negative sense RNA containing flanking sequences derived from IAV, was co-transfected along with the IAV genome segments. Cells were incubated for 48 h before the luciferase assay proceeded as previously described. For the segment 4 reporter assay, a plasmid encoding IAV segment 4 that is transcribed as a negative sense RNA was co-transfected in addition to the other IAV genome segments. For qRT-PCR experiments, cells were incubated for 24 h before RNA was extracted using TRI Reagent and the DNA removed using Turbo DNase (Thermo Fisher Scientific) followed by phenol/chloroform extraction. mRNA was amplified as described below for two-step qRT-PCR. Levels of positive sense segment 4 RNA were measured and normalized to GAPDH using the primers in Table 1.

**Table 1.** Primers used in qRT-PCR experiment

Primer	Forward	Reverse
GAPDH	AATCCCATCACCATCTTCCA	TGGACTCCACGACGTACTCA
IAV segment 4	TTGCTAAAACCCGGAGACAC	CCTGACGTATTTTGGGCACT

### TRIM25 RNA tethering assays

HEK TRIM25 KO cells were seeded in a 96-well plate at 15 000 cells per well for luciferase assays, or in a 24-well plate at 90 000 cells per well for RNA extractions. The following day the plate was transfected with psiCHECK2 containing nine MS2 stem loops in the 3′UTR of Renilla, or a construct without the nine MS2 stem loops and co-transfected with plasmids as indicated using Lipofectamine 2000 (Invitrogen). The nine stem loops were cloned in to psiCHECK2 by restriction digest from Luc-MS2 plasmids kindly gifted by Prof. Nicola Gray from the University of Edinburgh. The psiCHECK2 plasmid provides an internal control of Firefly luciferase, which is used for normalizing transfection efficiency and general effects on gene expression. The TRIM25-MS2 fusion protein was designed by linking the MS2 protein to the C-terminus of TRIM25 with a flexible linker (GSGGGGSRS) between. After 24 h, a luciferase assay was performed according to the instructions of the Dual-Luciferase Reporter Assay System kit (Promega) or the cells were resuspended in TRI Reagent (Sigma-Aldrich). For actinomycin D treatment the cells were transfected as above with psiCHECK2 containing nine MS2 stem loops and TRIM25-MS2 fusion protein. The following day Actinomycin D was added at a final concentration of 5 μg/ml and cells re-suspended in TRI Reagent at 0, 2, 4 and 6 h post treatment.

### qRT-PCR

RNA was extracted according to TRI reagent manufacturer's instructions. When indicated, DNase treatment was performed according to instructions for Turbo DNase (Thermo Fisher Scientific) and a phenol/chloroform extraction performed. Either GoTaq 1-Step RT-qPCR System (Promega) was used or reverse transcription for mRNAs was performed using Superscript IV RT (Thermo Fisher Scientific) and oligo(dT) (Thermo Fisher Scientific) and qPCR using GoTaq qPCR Master Mix (Promega) on a qTower (Analytik Jena). Primers used for Influenza segment quantification are listed in Table 2.

**Table 2.** Primers used in qRT-PCR experiment

Primer	Forward	Reverse
Segment 1	GTTGGGAGAAGAGCAACAGC	GATTCGCCCTATTGACGAAA
Segment 2	CGTACCGATGCCATAGAGGT	GGCTGACAAATGGGTTCAGT
Segment 3	ATGGGGAGGACCTGAAAATC	GCCCCTGTAGTGTTGCAAAT
Segment 4	TTGCTAAAACCCGGAGACAC	CCTGACGTATTTTGGGCACT
Segment 5	TGCTTCAAAACAGCCAAGTG	GCCCAGTACCTGCTTCTCAG
Segment 6	CCTGATACCGGCAAAGTGAT	CCCCACTGCAGATGTATCCT
Segment 7	CCTGGTATGTGCAACCTGTG	AGCCTGACTAGCAACCTCCA
Segment 8	CACGTGCTGGAAAGCAGATA	CTTATCCATGATCGCCTGGT

### Statistical analysis

All statistical tests applied are indicated in figures caption, as well as the number of samples analyzed. Data were expressed as mean ± standard deviation (*M* ± SD). A *P*-value <0.05 was considered statistically significant.

### Co-immunoprecipitation

HEK293 TRIM25 KO cells transfected with plasmids expressing T7-tagged TRIM25 and untagged ZAP were infected with the IAV PR8 R38K41A strain at an MOI of 5 for 6 h. The cells were disrupted by sonication and the extracts pre-cleared by incubation with 20 μl protein A agarose for 30 min. Anti-T7 antibody (69048 Merck Millipore) was coupled to 30 μl protein A dynabeads (Thermo Fisher Scientific) and incubated with the extracts for 1 h. The bound proteins were separated on a 4–12% SDS polyacrylamide gel and analyzed by western blotting. Washes of beads were done in Buffer G (20 mM Tris–HCl pH 7.5, 137 mM NaCl (Fisher Scientific), 1 mM EDTA, 1% Triton X-100 (Fisher Scientific), 10% glycerol, 1.5 mM MgCl_2_ (Fisher Scientific), 1 mM DTT, 0.2 mM PMSF (Thermo Fisher Scientific).

## RESULTS

### TRIM25 inhibits IAV replication irrespective of its RBD or RING domains

**Figure 1.**
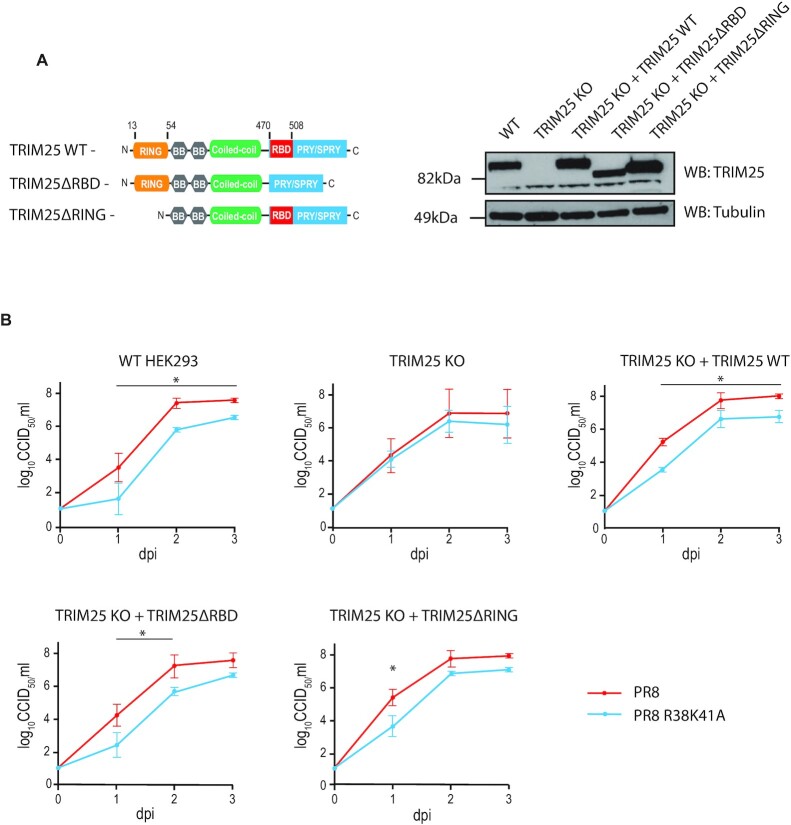
An IAV replication defect in TRIM25 KO HEK293 cells can be rescued with RNA binding or ubiquitination deficient TRIM25 mutants. (**A**) TRIM25 WT, TRIM25ΔRBD or TRIM25ΔRING were re-integrated into HEK293 TRIM25 KO cells using the Flp-In recombinase system. LHS: domain architecture of wild-type TRIM25 and deletion mutants. Relative position of host RNA-binding domain (RBD) is shown in red. RHS: levels of proteins were compared to WT cells by western blot. (**B**) Cell lines were infected with IAV PR8 WT or PR8 NS1 R38K41A (MOI = 0.0001) and virus titers were assessed by the endpoint dilution assay. Whiskers represent the standard deviation (SD) from three biological replicates. Dpi—days post-inoculation. The asterisk (*) indicates *P* < 0.05 in mixed-effect model followed by Sidak's multiple comparison test.

We have previously described the generation of a HeLa TRIM25 knock-out (HeLa TRIM25 KO) cell line through CRISPR/Cas9-mediated gene editing (16). To create a system to investigate TRIM25′s function, the same technique was used to generate a HEK293 TRIM25 knock-out cell line (TRIM25 KO) that could then be reconstituted with wild type or mutant TRIM25 genes (37). This cell line contains a flippase recognition target (FRT) site to allow stable integration of a gene of choice into the genome using the Flp-In recombinase (38). Codon optimized and T7-tagged TRIM25 WT, TRIM25ΔRBD (lacking RNA-binding residues 470–508 located in the PRY/SPRY domain) and TRIM25ΔRING, which does not have E3 ligase activity (lacking residues 13–54), were integrated into the genome at this site, resulting in stable expression at levels close to endogenous TRIM25 in HEK293 wild-type (WT) cells (Figure 1A). These cell lines were infected with either WT IAV A/Puerto Rico/8/34 (PR8) strain or a virus with R38A/K41A (R38K41A) mutations in NS1 that render it unable to inhibit TRIM25 and is defective in blocking the RIG-I pathway (23,31–34). Cells were infected at a low multiplicity of infection and viral replication was assessed by endpoint dilution assay (Figure 1B). In WT HEK293 cells, WT PR8 virus replicated better than the PR8 R38K41A mutant, presumably due to the presence of a fully active NS1 protein. In contrast, in HEK293 TRIM25 KO cells PR8 WT and R38K41A mutant grew to similar titers. Surprisingly, however, the growth defect of the mutant IAV was not only restored by integrated WT TRIM25, but also by TRIM25ΔRBD and by the integration of the ubiquitination deficient mutant TRIM25ΔRING (Figure 1B). TRIM25 KO cells grew more slowly than WT cells, and so cell-to-cell comparison with regards to IAV replication was not possible. Together, these results suggested that TRIM25′s anti-viral activity against IAV is not entirely dependent on its E3 ubiquitin ligase activity nor the part of the PRY/SPRY domain which binds RNA.

### Both TRIM25 and TRIM25ΔRBD bind to positive-sense IAV RNAs during infection

**Figure 2.**
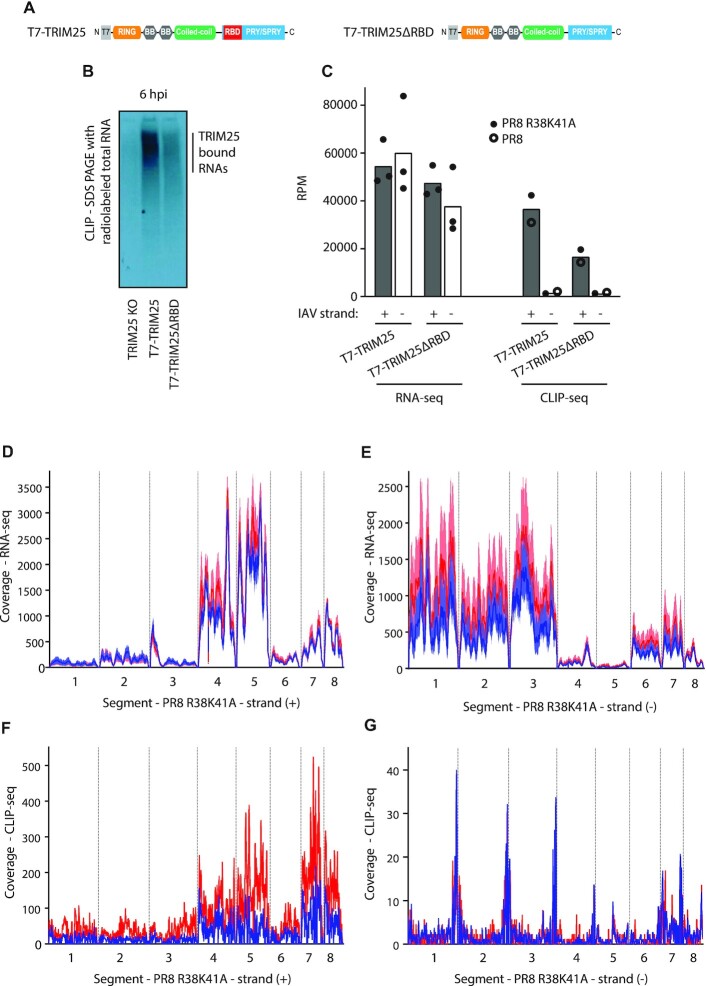
TRIM25 preferentially binds positive-sense IAV RNAs. (**A**) Domain architecture of wild-type TRIM25 and TRIM25ΔRBD tagged with T7. (**B**) T7-TRIM25 binds efficiently to RNA, while TRIM25ΔRBD RNA-binding is somewhat compromised, as seen on the SDS PAGE with 5′-end labeled, total RNA crosslinked and immunoprecipitated with anti-T7 Ab, 6 hpi (hours post infection) with the PR8 R38K41A IAV (MOI = 5). (**C**) RNA-seq analyses of HEK293 cells 6 hpi with the PR8 R38K41A IAV (MOI = 5) (filled circles) show comparable levels of (+) and (–) IAV strands, whereas CLIP-seq analyses of both T7-TRIM25 and T7-TRIM25ΔRBD infected with PR8 (empty circles) or PR8 R38K41A (filled circles) display preference for binding to (+) IAV strand RNAs. Bars represent geometric mean number of reads per million (RPM). (D, E) Coverage depth (reads) for the PR8 R38K41A IAV ((+) panel D, and (–) panel E) detected at 6 hpi with RNA-seq in T7-TRIM25 (red line) and T7-TRIM25ΔRBD cells (blue line). Line represents mean values (*n* = 3). Shaded area represents ranges. (F, G) Coverage depth (reads) for the PR8 R38K41A IAV ((+) panel F, and (–) panel G) detected 6 hpi with the CLIP-seq in T7-TRIM25 (red line) and T7-TRIM25ΔRBD cells (blue line). For analysis and presentation all segments were joined together. Coverage depth was adjusted per million of reads after quality trimming.

To test if TRIM25 binds IAV RNAs and to determine if the RNA-binding deficient TRIM25ΔRBD was still able to bind viral RNAs, we performed CLIP-seq as published previously (16). TRIM25 KO cells with integrated T7-tagged TRIM25 WT or TRIM25ΔRBD (Figure 2A) were infected with the IAV PR8 R38K41A strain at an MOI of 5 for 6 h followed by UV crosslinking (Figure 2B). TRIM25 bound more RNA than TRIM25ΔRBD, as seen on SDS PAGE with 5′-end labelled total RNA crosslinked and immunoprecipitated with anti-T7 Ab (Figure 2B, C). A CLIP-seq control using an irrelevant T7-tagged protein (eGFP) in uninfected HEK293 cells gave very little signal for 5′-end labelled RNA, indicating that RNA-binding was specific to the presence of TRIM25 (Supplementary Figure 1). TRIM25 was found to bind to all types of cellular RNAs but primarily to mRNAs, including viral ones (Supplementary Figure 2). There were no major differences in the relative amounts of RNA, viral or cellular bound after infection with WT PR8 compared to the R38K41A NS1 mutant (Supplementary Table S1).

To understand the specificity of TRIM25′s IAV RNA binding, we performed RNA-seq analysis after 6 h of infection of both TRIM25 WT and TRIM25ΔRBD with IAV PR8 R38K41A at an MOI of 5. This demonstrated that overall, both positive (+) and negative (−) strands of IAV were detected at similarly high levels (Figure 2C and Supplementary Table S1). Mapping of the individual reads to the virus genome showed that segment 4, 5, 7 and 8 mRNAs were detected more frequently that those from other segments, while vRNA levels of the polymerase genes (segments 1–3) were most abundant (Figure 2D, E), as expected (39). Crucially, TRIM25 CLIP-seq analyses predominantly identified TRIM25 binding on (+) strand viral reads, which could be either mRNAs or the replication intermediate cRNAs (Figure 2C, F). Surprisingly, both TRIM25 and TRIM25ΔRBD efficiently bound viral RNAs, which coincided with the RNA abundance, as seen by RNA-seq (Figure 2D, F). Viral (+) RNAs corresponding to segments 4, 5, 7 and 8 presented more pronounced TRIM25 binding than those of segments 1–3 and 6. This is most likely because RNAs from segments 4, 5, 7 and 8 are expressed at higher levels upon IAV infection than segments 1–3 and 6 (Figure 2D, F) and (39). The CLIP-seq interaction with (-) strands was much weaker and presented a pattern of association with untranslated regions (Figure 2G). A similar pattern of TRIM25 interactions was observed for PR8 WT IAV CLIP-seq analysis (Supplementary Figure 3). These results demonstrate that TRIM25 preferentially binds to (+) IAV strand with some binding at (−) segment extremities also present.

In CLIP-seq analysis, the positive sense strand of PR8 R38K41A segment 7 had the highest number of Reads Per Kilobase of transcripts per Million reads mapped (RPKM) at 6 h post infection both in WT (*n* = 29019) and TRIM25ΔRBD cells (*n* = 11269). Surprisingly, allocation of these reads measured upon infection moderately correlated (Spearman's ρ = 0.43) with the GpC (guanine-phosphate-cytosine) dinucleotide frequency in the IAV genome in the original HEK cells (Supplementary Figure 4), but not in modified TRIM25ΔRBD HEK cells (ρ = 0.11). On the contrary a lack of correlation was identified for the mirrored CpG dinucleotide both in original (ρ = −0.09) and modified HEK cells (ρ = 0.06). Other IAV segments had lower numbers of mapped reads and correspondingly a slightly lower correlation level was identified in the original HEK cells at 6 h post infection for the GpC dinucleotide (ρ = 0.33 for segment 5 and ρ = 0.41 for segment 8). This is in line with our previous data suggesting that TRIM25 prefers binding to RNA motifs rich in G and C (16).

These results indicate that TRIM25 preferentially binds positive-strand IAV RNAs. This could be because the (−) strand RNA is encapsidated by the IAV nucleoprotein (NP) as well as other viral and host factors, resulting in an impediment to TRIM25 binding.

### TRIM25 is not required for the IFN response to 5′ppp-RNA

**Figure 3.**
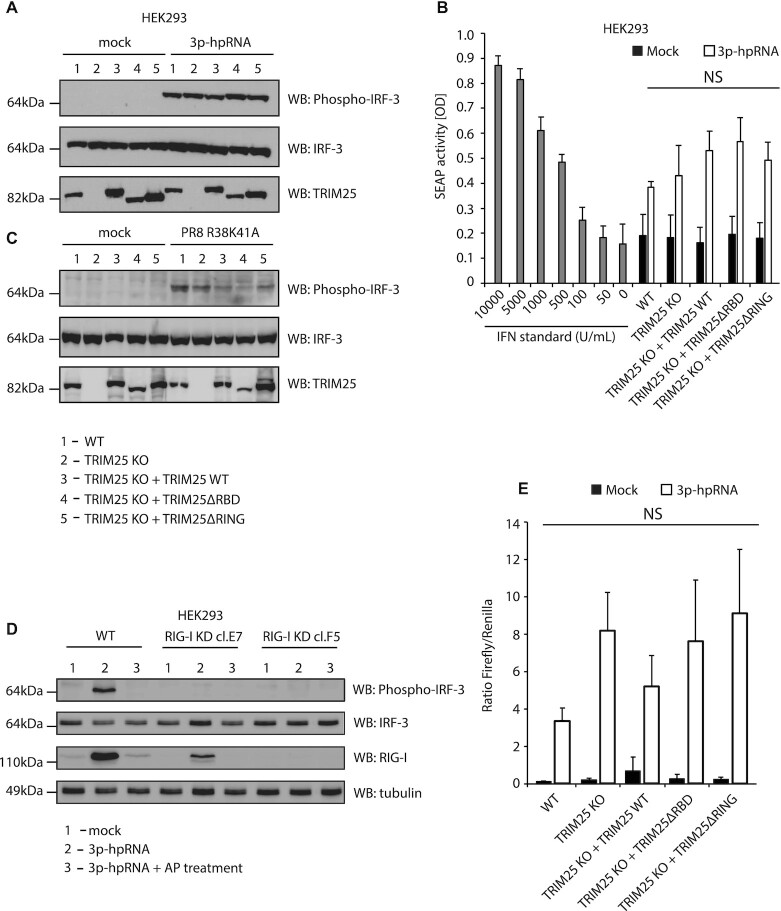
Deletion of TRIM25 from HEK293 cells does not affect activation of RIG-I signalling. (**A**) HEK293 cell lines were transfected with 100 ng/ml of the IAV-derived synthetic RNA 3p-hpRNA and incubated for 6 h. Levels of IRF-3 phosphorylation were assayed by western blot. (**B**) HEK293 cell lines were infected with PR8 R38K41A IAV (MOI = 15) and incubated for 24 h. Levels of IRF-3 phosphorylation were assayed by western blot. (**C**) HEK-Blue assay measuring secreted IFNα/β from each cell line 6 h after transfection with 100 ng/ml of 3p-hpRNA. The means and SDs of three independent experiments are shown. Statistical significance was calculated using one-way ANOVA with Tukey's post-test. (**D**) HEK293 cell lines expressing significantly lower levels of RIG-I were transfected with 100 ng/ml of the IAV-derived synthetic RNA 3p-hpRNA or dephosphorylated form of 3p-hpRNA and incubated for 6 h. Levels of IRF-3 phosphorylation were assayed by western blot. (**E**) Dual-luciferase assay measuring induction of the IFNβ promoter in each cell line 24 h after transfection with 100 ng/ml of 3p-hpRNA. Renilla luciferase was used as a loading and transfection control. The means and SDs of three independent experiments are shown. Statistical significance was calculated using one-way ANOVA with Tukey's post-test.

To assess the ability of the HEK293 cell lines bearing WT or mutant TRIM25 proteins to signal through RIG-I, they were transfected with a synthetic 5′ppp panhandle RNA (3p-hpRNA) derived from segment 8 of IAV which is known to induce signalling through RIG-I (40). Cells were transfected with 3p-hpRNA and incubated for 6 h before levels of IRF-3 phosphorylation were analysed by western blot (Figure 3A). There was no difference in activation of RIG-I signalling, as measured by IRF-3 phosphorylation, between the cell lines with wild type TRIM25, without TRIM25 or with stably integrated TRIM25ΔRING or TRIM25ΔRBD. Unchanged production of IFNα/β under the same experimental conditions was observed using the HEK-Blue bioassay system (Figure 3B). When the same cells were infected with PR8 R38K41A IAV at an MOI of 15, IRF-3 phosphorylation was slightly decreased in all mutant cell lines when compared with the HEK293 WT cells (Figure 3C). However, upon infection we could not detect production of IFNα/β (data not shown). To verify that the effects seen for the 3p-hpRNA were mediated by the RIG-I pathway, we generated CRISPR/Cas9 mutant cells that expressed significantly reduced levels of basal and IFN-induced RIG-I (Supplementary Figure 5) and used dephosphorylated forms of 3p-hpRNA treated with alkaline phosphatase (AP) that should not activate the RIG-I pathway. Indeed, RIG-I mutant cells transfected with 3p-hpRNA displayed no apparent IRF-3 phosphorylation when compared with the WT cells and AP-treated 3p-hpRNA also did not induce IRF-3 phosphorylation (Figure 3D). HEK-Blue bioassay confirmed complete shutdown of the RIG-I/ IFNα/β pathway in HEK293 RIG-I KD clone F5 (Supplementary Figure 6).

In addition, the activity of the IFNβ promoter in response to 3p-hpRNA transfection was assayed using a Firefly luciferase reporter construct. 3p-hpRNA was co-transfected into cells alongside the IFNβ promoter-Firefly plasmid and a plasmid constitutively expressing Renilla luciferase as a loading/transfection control and 24 h later levels of luciferase were assayed (Figure 3E). This assay also showed no significant difference in IFNβ promoter activity in cells that did not express TRIM25 compared to those that expressed wild type or mutant TRIM25. Taken together, these results strongly suggest that in HEK293 cells TRIM25 is not necessary for activation of RIG-I signalling, consistent with recent work indicating that RIPLET is the essential co-factor for RIG-I activation (14,15).

### RIG-I activation is reduced in the absence of TRIM25 in murine embryonic fibroblast cells (MEFs) but not in human HeLa and Calu-1 cell lines

**Figure 4.**
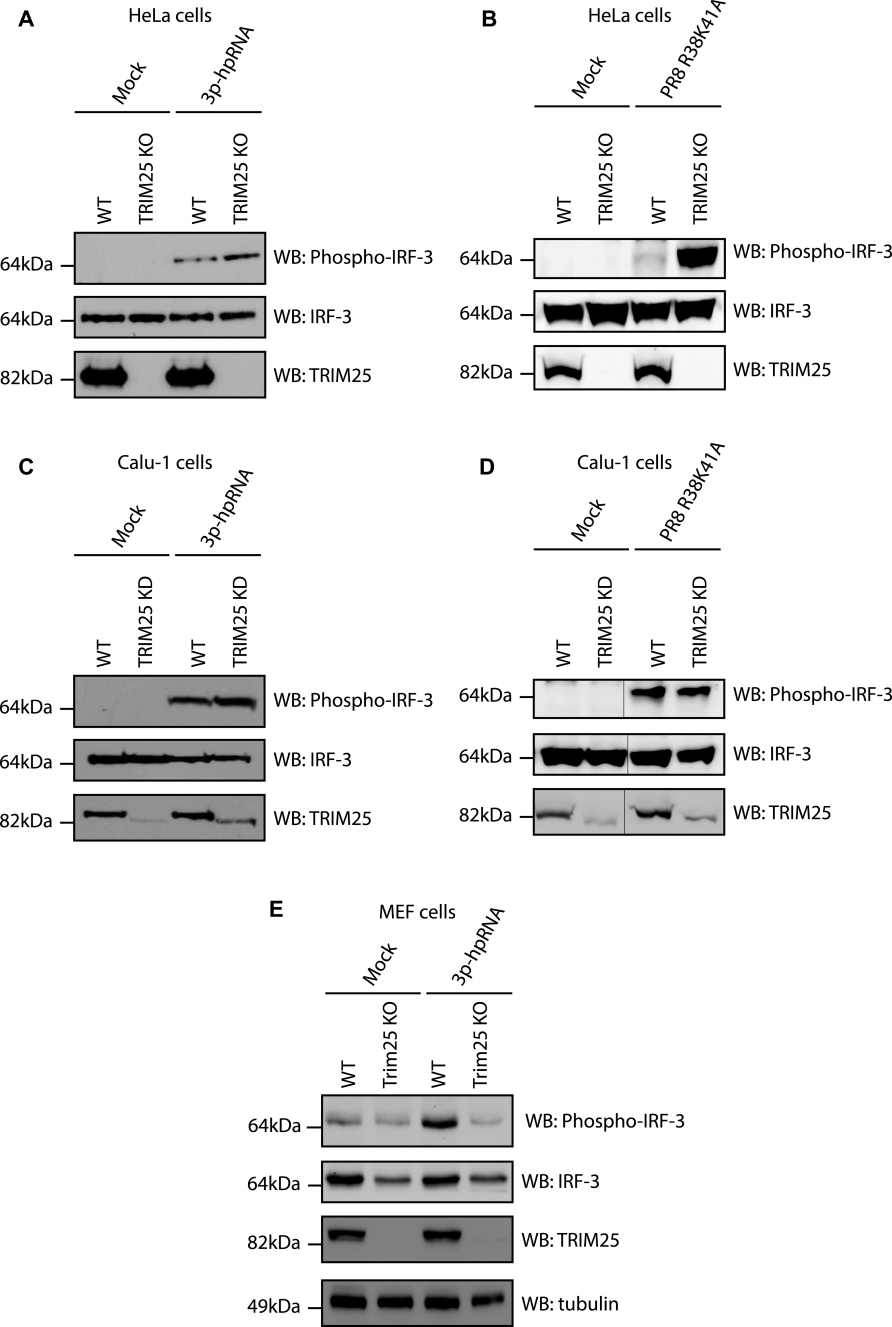
HeLa TRIM25 KO cells or Calu-1 TRIM25 KD, but not MEF TRIM25 KO maintain efficient phosphorylation of IRF-3 upon stimulation with 3p-hpRNA or IAV. (**A**) Western blot analysis of IRF-3 phosphorylation in HeLa WT and TRIM25 KO cells at 6 h post-transfection with 100 ng/ml 3p-hpRNA. (**B**) Western blot analysis of IRF-3 phosphorylation in HeLa WT and TRIM25 KO cells 24 hpi with PR8 R38K41A IAV (MOI = 15). (**C**) Western blot analysis of IRF-3 phosphorylation after 24 h in Calu-1 WT and TRIM25 KD cells transfected with 100 ng/ml 3p-hpRNA. (**D**) Western blot analysis of IRF-3 phosphorylation in Calu-1 WT and TRIM25 KD cells 24 hpi with PR8 R38K41A IAV (MOI = 15). (**E**) Western blot analysis of IRF-3 phosphorylation after 6 h in MEF WT and TRIM25 KO cells transfected with 100 ng/ml 3p-hpRNA.

To test whether an ability to activate RIG-I independently of TRIM25 was limited to HEK293 cells, RIG-I activation by 3p-hpRNA was assayed in other TRIM25 knock-out cell lines. Previously generated HeLa TRIM25 KO cells (16), in addition to HeLa wild type cells, were transfected with 3p-hpRNA and levels of phospho-IRF-3 were assayed by western blot after 6 h (Figure 4A). As seen in HEK293 cells, RIG-I/IRF-3 signalling was functional in both WT and TRIM25 KO HeLa cells; if anything, IRF-3 phosphorylation was more pronounced in HeLa TRIM25 KO cells. A similar phenotype was observed 24 h post infection with the PR8 R38K41A IAV at an MOI of 15 (Figure 4B). However, no induction of IFNα/β (measured by HEK Blue bioassay) was detected upon IAV infection in HeLa cells and thus we focused on the analysis of the first steps of RIG-I pathway activation. To further test the dependence of the RIG-I pathway on TRIM25, we used Calu-1 TRIM25 KD cells with one truncated TRIM25 allele left (RING domain and part CC domain) after CRISPR/Cas9 treatment (Figure 4C). This suggests that some expression of TRIM25 could be essential for Calu-1 growth. Nevertheless, the levels of the truncated TRIM25 were significantly lower than the WT protein in normal cells. Crucially, after both 3p-hpRNA transfection and infection with PR8 R38K41A IAV at an MOI of 15 the levels of IRF-3 phosphorylation were similar in WT and TRIM25 KD cells (Figure 4C, D). Finally, WT and TRIM25 KO mouse embryonic fibroblasts (MEFs—a gift from Prof. Jae Jung, University of Southern California (41)) were analysed for levels of phospho-IRF-3 in response to transfection of 3p-hpRNA after 6 h (Figure 4E). MEF cells showed detectable levels of phospho-IRF-3 in the absence of stimulation, but these were increased in WT cells by transfection of 3p-hpRNA. The levels of total IRF-3 were lower in MEF TRIM25 KO cells than WT cells, but the amount of phospho-IRF-3 was not increased by the 3p-hpRNA, suggesting that in this cell type, TRIM25 is involved in RIG-I activation.

Overall, these results suggest that RIG-I signalling can function efficiently in human HEK293, HeLa and Calu-1 but not mouse MEF cells in the absence of functional TRIM25. Thus, our results confirmed the dependence of RIG-I/interferon type I activation on TRIM25 in mouse cells (5) while providing further evidence that TRIM25 activity towards the RIG-I pathway is redundant in immortalized human cells. There is likely more variation between different species and even between cell lines of the same species as the role of TRIM25 may be defined by the presence of other proteins and cellular factors.

### The presence of TRIM25 does not restrict viral mRNA transcription

**Figure 5.**
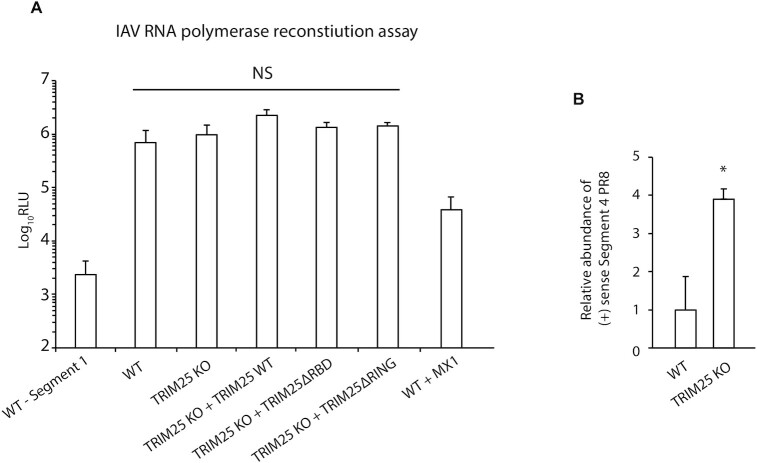
IAV RNA polymerase activity is insensitive to TRIM25 in HEK293 cells. (**A**) The indicated HEK293 cell lines were transfected with plasmids to reconstitute IAV RNPs as well as a reported plasmid encoding Firefly luciferase that is transcribed in the negative sense. Negative control experiments were performed in the same way but segment 1 (encoding PB2) of the IAV genome was omitted, preventing IAV RNA polymerase activity. The means and SDs of three independent experiments are shown. Statistical significance was calculated using one-way ANOVA with Tukey's post-test. (**B**) IAV RNPs were reconstituted along with segment four vRNA. Levels of positive sense segment four mRNA produced by the IAV polymerase were assayed by qRT-PCR and normalized to levels of GAPDH mRNA. The means and SDs of three independent experiments are shown. Statistical significance was calculated using Welch's *t*-test.

A previous study has shown that TRIM25 can inhibit transcription of IAV RNAs by blocking IAV’s polymerase activity (30). To test if this occurred in our cells, we performed a viral minireplicon assay. This involves transfecting cells with constructs expressing the components required for viral mRNA synthesis (polymerase subunits (PA, PB1, PB2) and NP) as well as a reporter plasmid encoding Firefly luciferase. Host machinery transcribes the Firefly luciferase gene into a negative sense RNA flanked by the 3′ and 5′ sequences of an IAV vRNA. For Firefly luciferase protein to be expressed, this negative sense RNA must be transcribed to a positive sense mRNA, a process that requires an active IAV RNA polymerase and therefore any inhibition of viral mRNA synthesis by TRIM25 can be measured. This assay was performed in the HEK293 cell lines and Firefly luciferase activity was determined (Figure 5A). Luciferase activity was not significantly different between WT, TRIM25 KO, TRIM25 KO + TRIM25ΔRBD or TRIM25 KO + TRIM25ΔRING cell lines, indicating that IAV RNA polymerase activity was not inhibited by the presence of TRIM25, at least for this luciferase reporter gene. Importantly, dynamin-like large GTPase MX1, a known inhibitor of IAV polymerase (42), efficiently inhibited luciferase production. Thus, in this system, TRIM25 did not detectably inhibit IAV gene expression.

To further test the effects of modulating TRIM25 expression, the polymerase assay was performed with segment 4 of the IAV genome, encoding the HA protein, as a reporter to see if TRIM25 inhibited IAV RNA polymerase activity on an authentic genome segment. The assay was performed in the same way as previously but transcription from negative to positive-sense mRNA and cRNA was analysed by qRT-PCR detecting positive sense RNA from segment 4 (Figure 5B). A fourfold difference in HA transcript production was seen between WT and TRIM25 KO cells, indicating that TRIM25 could be directly inhibiting IAV RNA polymerase activity or otherwise influencing IAV RNA abundance (Figure 5B). Taken together, these results suggest that TRIM25 inhibition of IAV is complex and could use yet unidentified molecular mechanisms.

### TRIM25 destabilizes IAV mRNAs during infection

**Figure 6.**
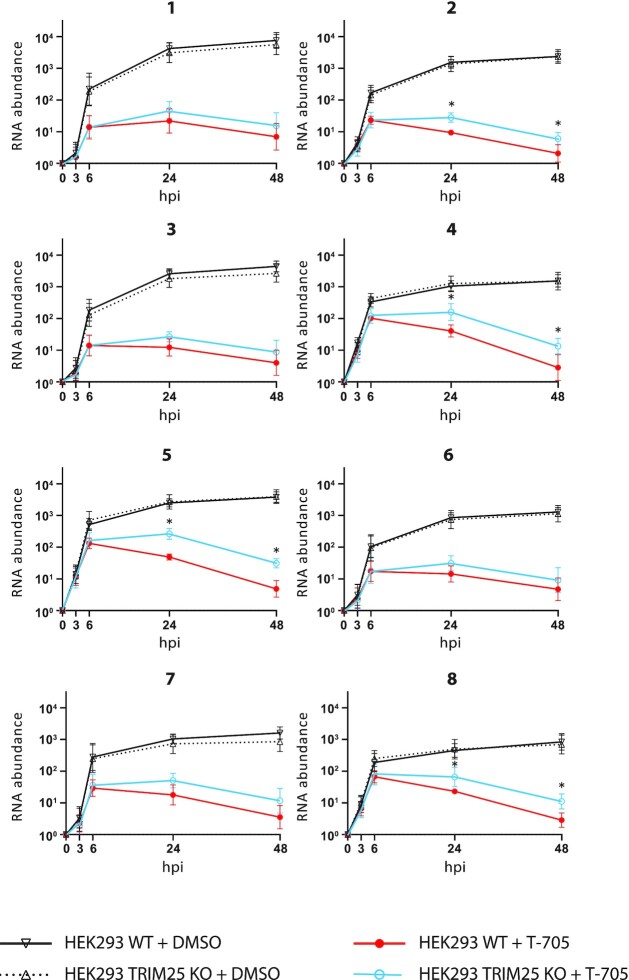
TRIM25 regulates stability of IAV RNA. HEK293 WT and HEK293 TRIM25 KO cells were infected with IAV PR8 R38K41A. After 30 min of infection cell were treated with Favipiravir (T-705) or DMSO (control) for 1.5 h. The relative changes in the amount of viral RNA segments (indicated with a number over the graphs) were measured using qRT-PCR in the cells at different time points. The level of RNA at 0 hpi was adjusted to one. Statistical significance was calculated upon log-transformation using two-way ANOVA with Sidak's post-test.

Since TRIM25 binds viral positive strand RNAs and restricts IAV PR8 infection, we investigated if this binding influenced the stability of IAV RNAs. We infected HEK293 WT and HEK293 TRIM25 KO cell with IAV PR8 R38K41A and then treated the cells with Favipiravir (T-705), a drug that inhibits the viral polymerase (43), and measured the changes in the amount of viral RNA segments (positive and negative combined), using qRT-PCR, in the cells at different time points. Of note previous reports showed that Favipriavir added only prior to infection allows for full IAV RNA synthesis inhibition (44), however, our experiments necessitated prior accumulation of IAV RNAs before the transcription inhibition to observe the effects of TRIM25 on the stability of IAV RNAs. Following Favipiravir treatment, viral RNAs were more stable in HEK293 TRIM25 KO cells than in HEK293 WT cells (Figure 6). While in control (DMSO-treated cells) there was no difference.

**Figure 7.**
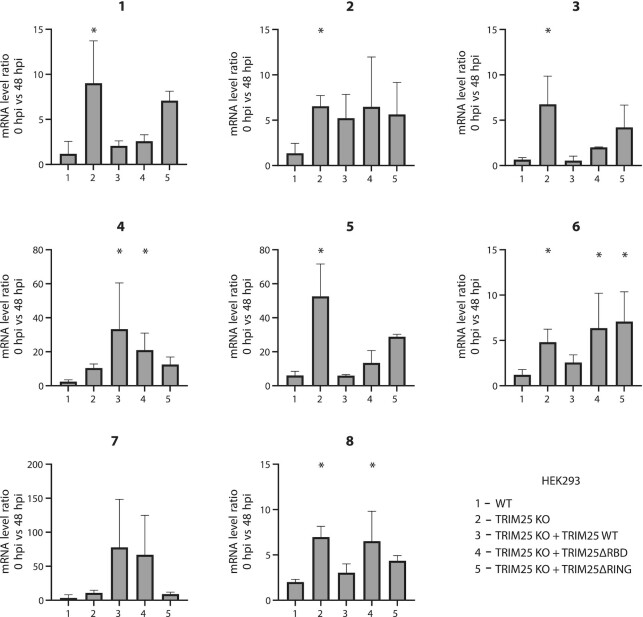
TRIM25 regulates the stability of IAV mRNAs. HEK293 WT, HEK293 TRIM25 KO, HEK293 TRIM25 KO + TRIM25 WT, HEK293 TRIM25 KO + TRIM25ΔRBD and HEK293 TRIM25 KO + TRIM25ΔRING were infected with IAV PR8 R38K41A and were treated with the viral polymerase inhibitor Favipiravir (T-705). The changes in the amount of viral mRNA segments were measured using qRT-PCR in the cells at 48 h after Favipiravir treatment. To quantify positive sense viral mRNAs a qRT-PCR with an oligo dT primer was used for the RT. Primers for specific viral segments were used for amplification of all 8 segments. The means and SDs of five biological replicates are shown. Statistical significance was calculated using ANOVA on ranks.

Since a standard qRT-PCR does not distinguish between the three different types of IAV RNAs (vRNA, mRNA, cRNA) a qRT-PCR using an oligo dT primer was used for the RT step to analyse viral mRNAs. Primers for specific viral segments were then used for the qPCRs step. This showed that at 48 h post treatment, the amount of mRNA from six out of eight segment-derived was significantly increased in the presence of Favipiravir in TRIM25 KO cells compared to WT cells (Figure 7). Cells expressing truncated TRIM25 constructs ΔRBD and ΔRING displayed efficient rescue of the TIRM25 KO phenotype for five out of eight segments (Figure 7). These results show that TRIM25 causes destabilization of some IAV mRNAs. Crucially, individual IAV mRNAs can associate with a plethora of other factors that could contribute or impede TRIM25 function, which might explain large variation in the obtained results, and warrant further investigations into the RNA-directed role of TRIM25 in viral infections.

### Direct tethering of TRIM25 to mRNA causes RNA degradation

**Figure 8.**
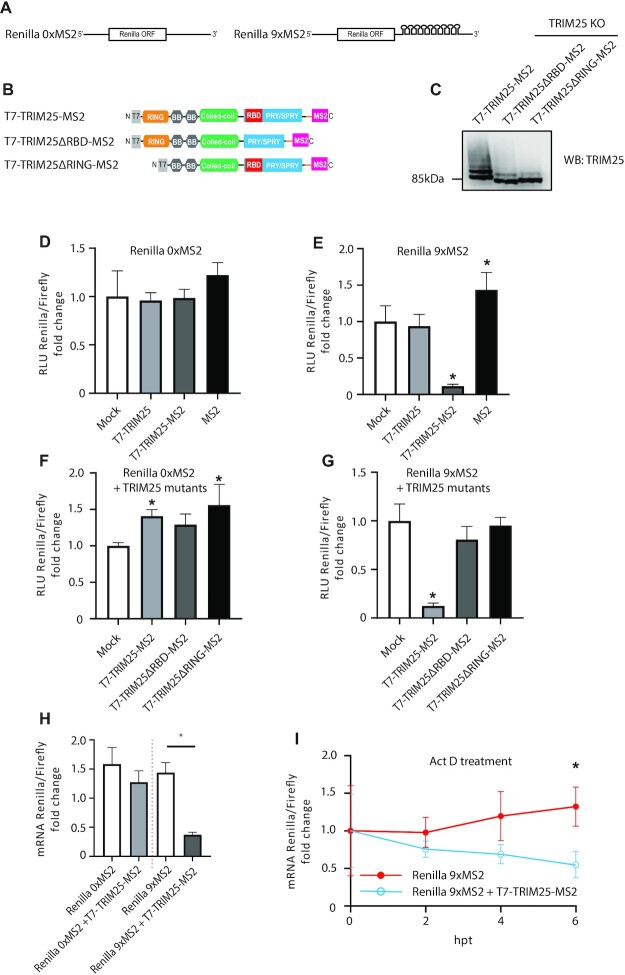
Tethering of TRIM25-MS2 to MS2 stem loops triggers mRNA degradation. (**A**) Sequence architecture of Renilla mRNA with and without nine MS2 stem loops located in the 3′UTR. (**B**) Domain architecture of fusion protein of TRIM25, TRIM25ΔRBD or TRIM25ΔRING with MS2 domain (which binds MS2 RNA stem loops), tagged with T7 epitope. (**C**) T7-TRIM25-MS2, T7-TRIM25ΔRBD-MS2 or T7-TRIM25ΔRING-MS2 were overexpressed in HEK293 TRIM25 KO cells and levels of the proteins were compared to WT cells by western blot. (D-E) The psiCHECK2 plasmids without or with nine MS2 stem loops were co-transfected with plasmids expressing T7-TRIM25 WT, T7-TRIM25-MS2 fusion protein or MS2 on its own in to HEK293 TRIM25 KO cells and analysed with dual luciferase assay. (**F**, **G**) T7-TRIM25-MS2, T7-TRIM25ΔRBD-MS2 and T7-TRIM25ΔRING-MS2 fusion proteins were co-transfected with the psiCHECK2 constructs in to HEK293 TRIM25 KO cells and processed with dual luciferase assay. (**H**) Renilla mRNA levels were assayed upon reverse transcription, followed by DNase treatment and qPCR. Renilla mRNA with and without nine MS2 stem loops was measured after mock or T7-TRIM25-MS2 overexpression. (**I**) Actinomycin D treatment was preformed after co-expressing the psiCHECK2 plasmid containing nine MS2 stem loops with T7-TRIM25-MS2 fusion protein. Renilla mRNA levels were measured upon reverse transcription, followed by DNase treatment and qPCR at 0, 2, 4 and 6 h following actinomycin D treatment. The means and SDs of three (D, E, H, I) or six (F, G) independent experiments are shown. Statistical significance was calculated using one-way ANOVA with Dunnett's (D, E) or Sidak's (**H**) post-test, two-way ANOVA with Sidak's post-test (**I**) and ANOVA on ranks (F, G) The asterisk (*) indicates *P* < 0.05.

To further examine TRIM25′s effect on mRNA stability and to test if this is a direct or indirect mechanism, we tethered TRIM25 to Renilla luciferase mRNA. To do so, we introduced nine MS2 stem loops into the 3′UTR of Renilla luciferase mRNA encoded in a psiCHECK2 plasmid, a dual luciferase construct which also encodes firefly luciferase as a separate transcriptional unit. This plasmid, or a version without MS2-binding sites on the Renilla luciferase gene (Figure 8A) was co-transfected into TRIM25 KO HEK293 along with plasmids expressing the various TRIM25-MS2 fusion proteins (Figure 8B) or untagged TRIM25 or MS2 on its own and the ratio of the two types of luciferase activity measured to give a relative value of their expression efficiency. Western blot analysis confirmed equal expression of the TRIM25-MS2 fusion proteins (Figure 8C) and expression of any of these polypeptides alongside a luciferase gene lacking an MS2-binding site had no effect on Renilla activity levels (Figure 8D). In contrast, when an MS2-tagged Renilla luciferase mRNA was expressed, the T7-TRIM25-MS2 fusion protein significantly reduced (*P* = 0.0008) Renilla luciferase levels, while MS2 alone or a TRIM25 lacking the MS2 binding domain did not (Figure 8E). This provided direct evidence that attracting TRIM25 towards a specific mRNA reduced its expression. To explore the importance of RNA binding and ubiquitination on TRIM25′s ability to destabilize mRNAs, we co-transfected T7-TRIM25ΔRBD-MS2 and T7-TRIM25ΔRING-MS2 fusion proteins with the psiCHECK2 constructs. For reasons that are yet unclear, co-expression of untagged WT TRIM25 or the MS2-tagged ΔRING construct caused slight but statistically significant increases in relative levels of Renilla luciferase expression from the mRNA lacking MS2-binding sites (Figure 8F). However, neither mutant TRIM25 polypeptide significantly inhibited expression of the tagged mRNA (Figure 8G), indicating that both RBD and RING domains are necessary for this activity.

To test whether TRIM25 influenced Renilla mRNA levels when tethered to it (rather than, for instance, by reducing mRNA translation), we measured Renilla transcript levels by qRT-PCR after DNase treatment (Figure 8H). Importantly, we saw a significant decrease in Renilla mRNA levels only when co-expressing the psiCHECK2 construct containing nine MS2 stem loops and T7-TRIM25-MS2 fusion protein, confirming that TRIM25 acts at the transcript level. Treatment with the RNA polymerase II inhibitor Actinomycin D confirmed that TRIM25 affected mRNA stability, since levels of the Renilla mRNA bearing MS2 stem loops decreased significantly faster after treatment with the drug when co-expressed with T7-TRIM25-MS2 fusion protein (Figure 8I).

## DISCUSSION

**Figure 9.**
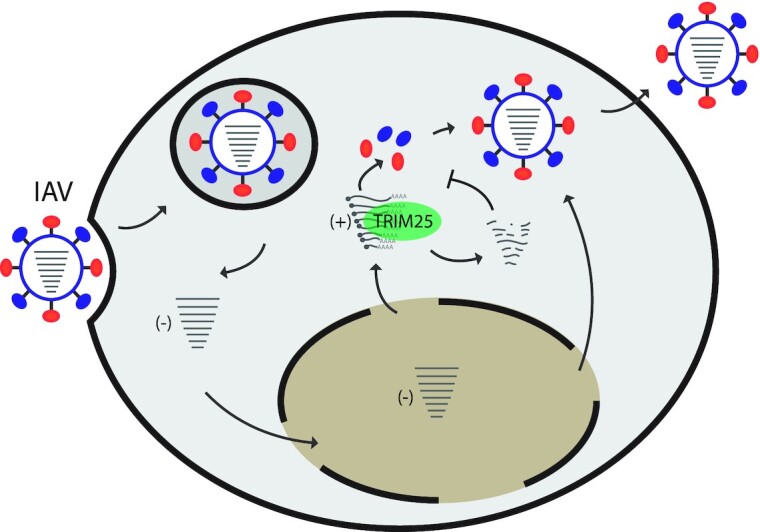
Model of TRIM25 sensing and inhibiting IAV infection by controlling viral mRNA stability. During IAV infection, TRIM25 binds to positive strand RNAs and triggers mRNA degradation, which in turn might be one of the factors that contributes to inhibition of viral replication. Our results also predict additional, E3 ligase-independent mechanisms of TRIM25-mediated control of IAV infection.

Here, we have investigated the antiviral mechanisms of TRIM25 and identified a RIG-I independent mode of action. We show that deletion of TRIM25 from HEK293 cells relieved inhibition of an NS1 mutant of IAV unable to fully control innate immune responses. As would be expected, inhibition was restored by re-integration of TRIM25 WT, but surprisingly, the RNA binding-deficient mutant TRIM25ΔRBD and the ubiquitination activity-deficient TRIM25ΔRING also rescued the TRIM25 KO phenotype. This suggests that TRIM25 may perform another role in restricting IAV not involving its E3 ubiquitin ligase activity. Interestingly, our CLIP-seq results revealed that TRIM25ΔRBD could still bind viral RNAs, using a yet unknown mechanism, indicating that RNA binding could be important for TRIM25′s function. To comprehensively show that, construction of a TRIM25 mutant that had completely lost its RNA binding ability while preserving its E3 ubiquitin ligase function would be necessary. We also found that deletion of TRIM25 did not significantly affect the RIG-I response to an IAV-derived 5′ppp-RNA in these same cells, contrary to previously described results (6,45), but in line with more recent reports showing that RIPLET, rather than TRIM25, is critical for RIG-I activation in human cells (14,15). The RIG-I response is attenuated by TRIM25 knockout in MEF cells, showing that the role and necessity of TRIM25 may vary in the context of different cell types and organisms. Similar but smaller effect on the IFNβ expression was seen in the TRIM25 KO MEF cells exposed to 42bp dsRNA with 5′ppp (14). We further show that human TRIM25 does not directly inhibit transcription of IAV vRNAs in HEK293 cells, as has been shown in a previous study (30). Here for the first time, we show that TRIM25 binding to viral mRNAs triggers their degradation, and our proposed model is illustrated (Figure 9). These observations were reinforced with an RNA tethering experiment where TRIM25 directly inhibited levels of bound mRNA. We note that RNA stability assays with MS2 stem loops, unlike IAV infections indicated the need for RING and RBD domains. We thus speculate that TRIM25 might have other RING and RBD-independent functions that are crucial for its role in inhibiting viral infection.

The exact mechanism of TRIM25-mediated degradation of IAV mRNAs and its extent to endogenous RNAs or RNAs produced by other viruses should be studied next. Nonetheless, TRIM25 is known to interact with the zinc-finger antiviral protein (ZAP), which has been shown to degrade viral RNAs (reviewed in ^(^46^), (^47^)^). In addition, TRIM25 has been shown to be required for ZAP’s antiviral function, however, the exact mechanism for this remains unclear (48,49). We also detected efficient co-IP and colocalization of TRIM25 and ZAP which was not affected by the presence or absence of IAV infection (Supplementary Figure 7). It is nevertheless plausible that ZAP binds to RNA and uses TRIM25 to trigger RNA degradation. Intriguingly, our previous results showed that in undifferentiated mouse cells TRIM25 helps trigger exoribonuclease DIS3L2-mediated degradation of the let-7 microRNA precursor (21). It remains to be established whether these or other factors are responsible for TRIM25-mediated degradation of viral mRNAs. Furthermore, while ZAP was found to bind CpG dinucleotides on viral RNAs (50), our CLIP-seq analysis does not reveal this preference for TRIM25 on IAV mRNA, with GpC dinucleotides preferred. Intriguingly, a clear preference for the GpC dinucleotide was observed for TRIM25 but not for TRIM25ΔRBD. This could be explained by alternative RNA-binding domains providing broader sequence specificity for TRIM25. Indeed, such domains have been already identified, including a 7K motif, situated between coiled coil and the PRY/SPRY domain (20). Finally, TRIM25 has been shown to aid ZAP’s RNA binding ability, perhaps to overcome the CpG dinucleotide suppression in viral RNAs (51,52). The interactions of TRIM25 and ZAP during viral infection and the effect on the stability of viral mRNAs and virus replication needs to be further investigated.

Our results demonstrate for the first time that TRIM25 binds IAV mRNA and triggers its degradation. This provides an additional mechanism by which TRIM25 can restrict IAV infection. It is possible that TRIM25′s RNA binding role is important in the context of other RNA viruses. Thus, uncovering the mechanism and TRIM25′s protein binding partners used in its RNA degrading role will be of great importance. In summary, our results reveal novel, unexpected features of TRIM25′s roles in innate immune antiviral pathways and could help designing innovative, host-based RNA virus-targeted therapies.

## DATA AVAILABILITY

The accession number for RNA-seq and CLIP-seq data is PRJNA810715 in NCBI BioProject.

## Supplementary Material

gkac512_Supplemental_File

## Notes

Present address: Rute Maria Dos Santos Pinto, MRC-University of Glasgow Centre for Virus Research, Glasgow, UK.
