# Supplementary material for: TRIM25 inhibits influenza A virus infection, destabilizes viral mRNA, but is redundant for activating the RIG-I pathway
Source: Nucleic Acids Res. 2022 Jun 23;50(12):7097–114. doi: 10.1093/nar/gkac512 (PMC9262604; doi:10.1093/nar/gkac512)
Supplement: gkac512_Supplemental_File [file gkac512_supplemental_file.docx]

**Supplementary Figure 1. T7-TRIM25 binds efficiently to RNA**, while TRIM25ΔRBD RNA-binding is compromised, as seen on the SDS PAGE with 5′-end labeled, total RNA crosslinked and immunoprecipitated with anti-T7 Ab.

**Supplementary Figure 2. Distribution of CLIP-seq reads (adjusted to reads per million – RPM) for TRIM25 WT and TRIM25ΔRBD in cells infected with either PR8 (left panel) or PR8 R38K41A IAV (right panel).**

**Supplementary Figure 3. Coverage depth (reads) for the PR8 IAV ((+) left panel and (-) right panel) detected 6 hours post infection with CLIP-seq in HEK293 T7-TRIM25 WT (red line) and HEK293 T7-TRIM25ΔRBD cells (blue line).** Coverage depth was adjusted per million of reads after quality trimming.

**
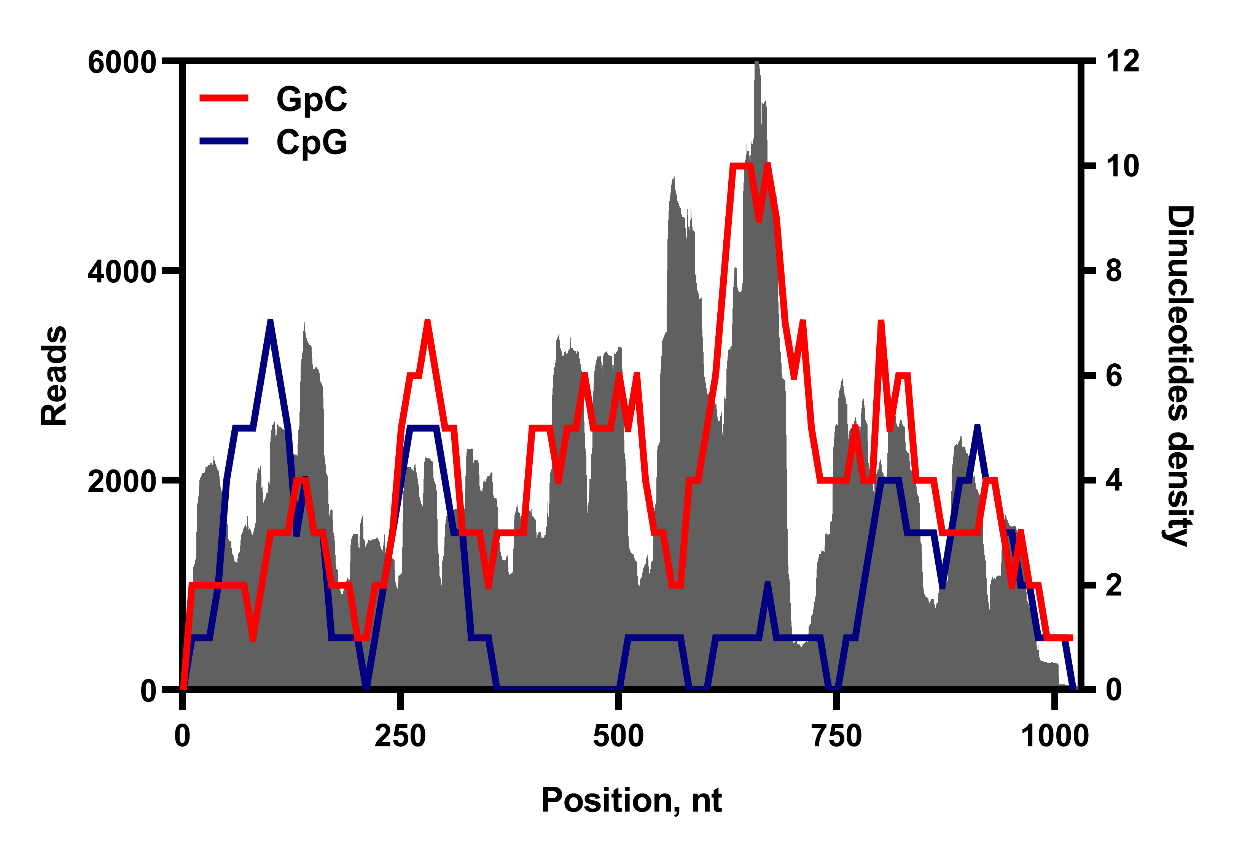
**

**Supplementary Figure 4. Correlation of mapped reads with specific dinucleotide density.** Grey shaded area represents absolute numbers (left Y-axis) of mapped in CLIP-seq reads for segment 7 of the IAV genome at 6 hpi in HEK293 TRIM25 KO cells with T7-TRIM25. An R script (https://github.com/itrus/Mapping-ROIs-in-genetic-sequences) was used to count dinucleotides in a 70-nucleotide sliding window. Dinucleotide frequencies are represented with lines (secondary Y-axis).

**
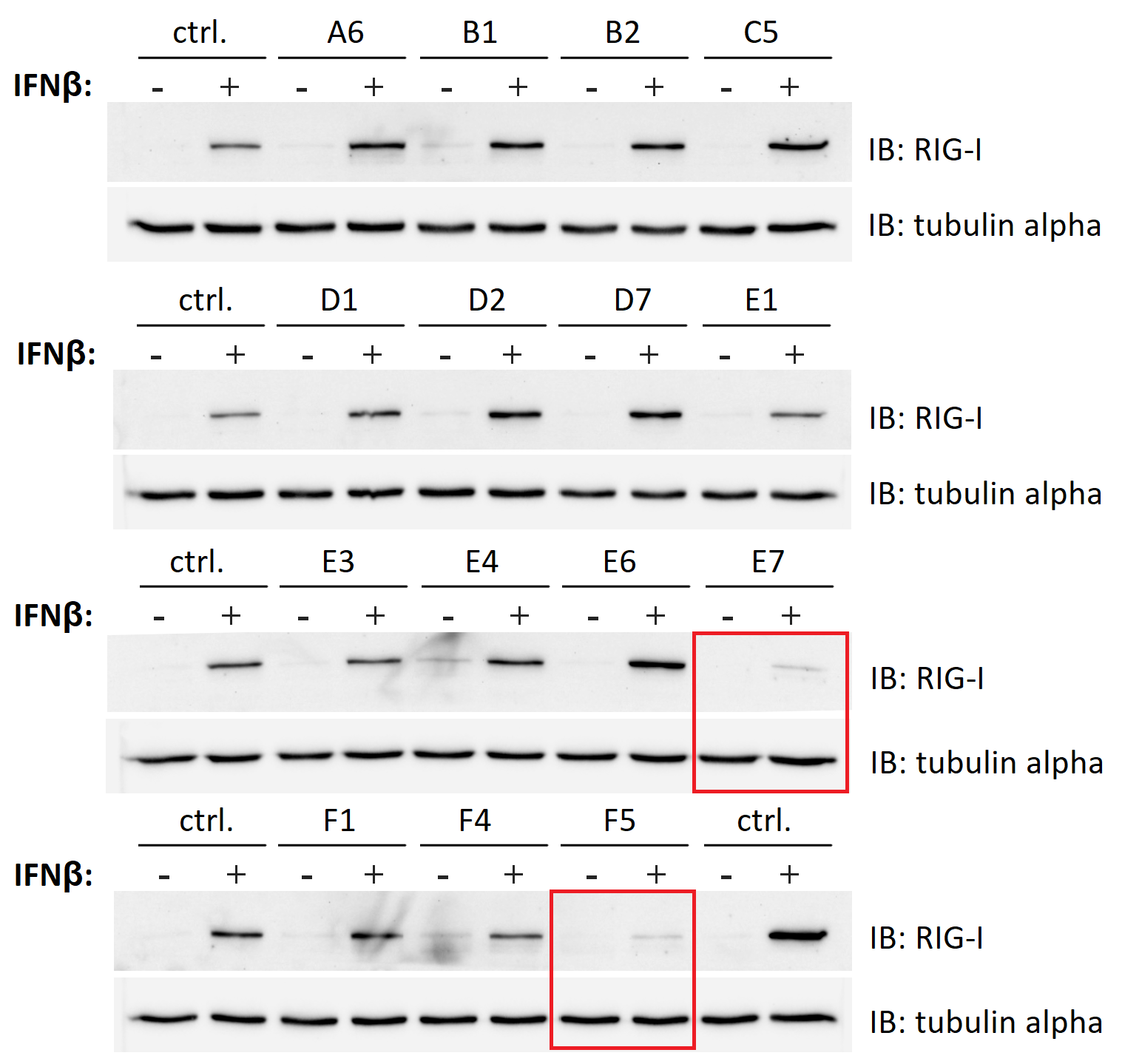
**

**Supplementary Figure 5. Generation of RIG-I depleted HEK293 cells using CRISPR/Cas9.** Western blot analyses of RIG-I and tubulin alpha controls are shown. Where indicated (+) cells were stimulated with recombinant human interferon beta (20 ng/ml) for 18 hrs before analysis. Clones E7 and F5 were chosen as the ones expressing significantly lower basal and activated levels of RIG-I.

**Supplementary Figure 6. Interferon beta stimulation by 3p-hpRNA is dependent on RIG-I.** HEK-Blue assay of supernatants from HEK293 WT and paired RIG-I KD cells 24 hours post-transfection with 100 ng/mL 3p-hpRNA (untreated or pre-treated with alkaline phosphatase – AP). The means and SDs of four independent experiments are shown. Statistical significance (asterisks) was calculated using ANOVA on ranks.

**Supplementary Figure 7. ZAP and TRIM25 interact in uninfected and PR8 R38K41A infected cells.** (A) Western blot analysis of co-IP of overexpressed ZAP-L with T7-TRIM25 shows efficient interactions with and without infection with PR8 R38K41A virus 6 hpi MOI = 5. (B) Representative confocal photomicrograph illustrating mock- and PR8 R38K41A IAV-infected (MOI = 15) HEK293 cells. Cell nuclei were stained with Hoechst blue (10 μg/ml of Hoechst 33342). Red fluorescence visualises ZAP antigen (1/180 of Sigma HPA059096-25UL and 1/500 of Thermo A21244). TRIM25 is visualized by green fluorescence (1/160 Thermo MA5-41081 and 1/200 of Thermo F2761). Scale bar represents 5 μm.

Supplementary Table 1 – Sequencing results for the specimens related to Figure 2.

| **Sample identification** | | | | | | **Number of raw reads** | **Number of quality-trimmed reads** | **Reads mapped to host genome with Kallisto** | | **Reads mapped to IAV genome with bowtie2** | | | | **Ratio of reads mapped to IAV vs. host** |
| --- | --- | --- | --- | --- | --- | --- | --- | --- | --- | --- | --- | --- | --- | --- |
| **Analysis** | **Virus** | **BioSample accession number** | **Cell** | **Hours post inoculation** | **Replicate** |  |  | **Total number of reads** | **Percentage of reads mapped to host** | **Reads mapped to positive-sense genome** | **Reads mapped to negative-sense genome** | **Total number of reads** | **Percentage of reads mapped to IAV** |  |
| RNA-seq | PR8 R38K41A | SAMN26275652 | HEK-WT | 6 | 1 | 19,523,147 | 19,122,442 | 10,203,175 | 53.36% | 1,109,677 | 882,446 | 1,992,123 | 10.42% | 19.52% |
|  |  | SAMN26275653 | HEK-WT | 6 | 2 | 20,371,582 | 19,913,830 | 10,131,819 | 50.88% | 838,969 | 1,452,384 | 2,291,353 | 11.51% | 22.62% |
|  |  | SAMN26275654 | HEK-WT | 6 | 3 | 19,648,154 | 19,150,508 | 9,914,097 | 51.77% | 841,565 | 756,166 | 1,597,731 | 8.34% | 16.12% |
|  |  | SAMN26275649 | HEK-RBD | 6 | 1 | 26,275,680 | 25,545,904 | 13,541,610 | 53.01% | 946,894 | 692,713 | 1,639,607 | 6.42% | 12.11% |
|  |  | SAMN26275650 | HEK-RBD | 6 | 2 | 18,782,762 | 18,355,024 | 9,923,832 | 54.07% | 884,286 | 458,248 | 1,342,534 | 7.31% | 13.53% |
|  |  | SAMN26275651 | HEK-RBD | 6 | 3 | 28,327,228 | 27,709,159 | 14,745,341 | 53.21% | 1,064,012 | 1,288,975 | 2,352,987 | 8.49% | 15.96% |
| CLIP-seq |  | SAMN26275640 | HEK-KO | 1 | 1 | 1,109,521 | 794,310 | 220,446 | 27.75% | 19 | 6 | 25 | 0.00% | 0.01% |
|  |  | SAMN26275642 | HEK-WT | 1 | 1 | 3,517,524 | 2,796,639 | 479,155 | 17.13% | 83 | 10 | 93 | 0.00% | 0.02% |
|  |  | SAMN26275641 | HEK-RBD | 1 | 1 | 3,162,063 | 2,260,373 | 590,873 | 26.14% | 79 | 14 | 93 | 0.00% | 0.02% |
|  |  | SAMN26275643 | HEK-KO | 6 | 1 | 427,754 | 328,104 | 89,718 | 27.34% | 2,103 | 139 | 2,242 | 0.68% | 2.50% |
|  |  | SAMN26275645 | HEK-WT | 6 | 1 | 2,413,001 | 2,238,546 | 816,225 | 36.46% | 37,484 | 1,146 | 38,630 | 1.73% | 4.73% |
|  |  | SAMN26275644 | HEK-RBD | 6 | 1 | 4,504,231 | 4,307,818 | 1,512,452 | 35.11% | 36,077 | 2,517 | 38,594 | 0.90% | 2.55% |
|  | PR8 | SAMN26275646 | HEK-KO | 6 | 1 | 345,493 | 271,472 | 74,552 | 27.46% | 1,863 | 139 | 2,002 | 0.74% | 2.69% |
|  |  | SAMN26275647 | HEK-WT | 6 | 1 | 1,067,686 | 994,293 | 376,968 | 37.91% | 15,018 | 1,018 | 16,036 | 1.61% | 4.25% |
|  |  | SAMN26275648 | HEK-RBD | 6 | 1 | 3,284,454 | 3,107,818 | 1,047,985 | 33.72% | 21,792 | 2,658 | 24,450 | 0.79% | 2.33% |
